# Implicit Goalkeeper Influences on Goal Side Selection in Representative Penalty Kicking Tasks

**DOI:** 10.1371/journal.pone.0135423

**Published:** 2015-08-12

**Authors:** Benjamin Noël, John van der Kamp, Daniel Memmert

**Affiliations:** 1 Institute of Cognitive and Team/Racket Sport Research, German Sport University Cologne, Cologne, Germany; 2 Research Institute MOVE, Faculty of Human Movement Sciences, VU University Amsterdam, Amsterdam, The Netherlands; 3 Institute of Human Performance, University of Hong Kong, Hong Kong, China; University of Turin and the Italian Institute of Technology, ITALY

## Abstract

In well-controlled lab situations, marginal displacements of the goalkeeper on the goal line affect goal side selection of penalty takers implicitly, that is, without the penalty takers being consciously aware of the displacement. Whether this effect is retained in more representative real-life situations with competing goalkeepers and penalty takers has not been verified. In the current study, penalty takers were instructed to position the goalkeepers at the centre of the goal. They then performed penalty kicks adopting either a keeper independent or a keeper dependent strategy, while goalkeepers actually attempted to save the ball by strategically diving early or late. Analyses of trials in which penalty takers failed to place the goalkeeper in the centre of the goal (although they incorrectly believed they placed the goalkeeper at the centre of the goal) showed that implicit influences of the goalkeeper’s position on goal side selection were overridden by the (conscious) perception of the direction of the goalkeeper’s dive, but only if the penalty takers deliberately monitored the goalkeeper’s action *and* the goalkeeper committed early enough for penalty takers to respond. In all other combinations of penalty kick and goalkeeper strategies more than 60% of the kicks were directed to the side of the goal with more space. Most importantly, however, the current study shows that influences of implicit perception on the penalty takers’ decision making are rather pervasive considering that many supraliminal sources of information were available. That is, the current study demonstrates that implicit perception retains its influence on decision-making even if other (stronger) stimuli are also present.

## Introduction

In soccer penalty kicking, a goalkeeper positioned marginally to one side of the centre of the goal affects a penalty taker’s choice to what side to kick the ball, even though the penalty taker is not consciously aware of the goalkeeper standing off-centre. That is, in cases goalkeepers stand marginally to the left or right of the goal’s centre penalty takers direct about 60% of the kicks to the side of the goal with greater space. This so-called off-centre effect applies to penalty kicks taken against schematic computer animations [1] and more photorealistic goalkeepers [2, 3]. In addition, analyses of penalty kicks taken during professional competitions (e.g., FIFA World Cup, UEFA Champions League and the English Premier League) confirmed that the ball is more often directed to the side with more space, albeit that these observations cannot rule out that players acted strategically [1].

Research so far, however, has not verified whether the off-centre effect actually occurs in experimental situations that are more representative for competitive penalty kicks. In this regard research on anticipation in soccer penalty kicks has neatly illustrated that perception and action differs between video simulation and real life or in-situ tasks [4, 5]. For example, the gaze patterns of goalkeepers in laboratory-based set-ups using video presentations (e.g., of a penalty taker kicking the ball) are critically different from their gaze patterns in real-life situations where they attempt to actually intercept a ball [6]. Consequently, findings from laboratory-based experiments are not always readily applicable to real-life situations, and recommendations for sport players should only be made after laboratory-based findings are verified in more representative, real-life situations.

Accordingly, one may wonder whether the off-centre effect would survive on a soccer pitch, which contrary to the laboratory provides a vast array of information. Moreover, it remains to be seen if the implicit influence of goalkeeper position on the kicker’s goal side selection (i.e., that kickers tend to chose the side with greater space, even though they are not consciously aware of the goalkeeper standing off-centre) is retained when the kicker is actually confronted with a goalkeeper who acts in order to save the ball, instead of a static picture of a goalkeeper (as per previous studies). Addressing the off-centre effect in more representative, real-life situations can have important ramifications for goalkeepers who consider exploiting the effect in competition (by positioning themselves marginally off-centre), but will also help understanding fundamental issues as to what degree subliminal or implicit perception effects are interrupted or overridden by conflicting, more explicit perceptions.

Previous research has demonstrated that the strategies adopted by goalkeepers and penalty takers [7, 8] can have a major impact on the outcome of the penalty kick. Particularly, strategic decisions whether or not to take the opponent’s actions into account may overrule any effects of implicit perception of the goalkeeper’s position. Goalkeepers typically chose one of two strategies [9]. They can prioritize timing by anticipating a penalty taker’s intentions and moving before foot-ball contact to assure that they are in time. By doing so, they run the risk of choosing the wrong side, because information available from the kicker’s movements is not fully reliable [6, 10, 11]. Alternatively, goalkeepers can prioritize direction and choose to move after foot-ball contact to ascertain that they jump to the correct side, but then they run the risk to be (too) late. Penalty takers also select one of two strategies [8]. They can either decide on a kick direction before the run-up and uphold that decision irrespective of a goalkeeper’s actions during the run-up (keeper independent strategy), or they can choose a provisional side at the start of the run-up but anticipate the goalkeeper’s actions and–if demanded–adjust their decision to direct the ball to the side of the goal the goalkeeper is not defending (keeper dependent strategy). Importantly, the two penalty kick strategies differ with regard to the allocation of attention they induce [7]. Penalty takers using the keeper dependent strategy visually attend to the goalkeeper throughout almost the entire run-up, whereas penalty takers adopting the keeper independent strategy only attend to the goalkeeper in preparation for the run up, but then focus on the ball [7, 12, 13]. Consequently, in the keeper dependent strategy provisional decisions on kick direction are continuously updated based on current information about a goalkeeper’s intentions and actions, while in the keeper independent strategy these decisions predominantly rely on information about a goalkeeper before the run up (e.g., position on the goal line, and perhaps known directional preferences). Hence, penalty takers adopting a keeper independent strategy–and recent observations suggest that the vast majority of professional soccer players do so [14]–may be more liable to the off-centre effect than penalty takers who follow a keeper dependent strategy. That is, for a kicker employing a keeper dependent strategy, a goalkeeper who dives early (approx. 400 ms before football-contact [8, 15]) provides crucial information (and time) for updating decisions on kick direction, possibly overruling any implicit influences of the goalkeeper’s position before his dive. However, in case the goalkeeper dives to one side late, even penalty takers that employ a keeper dependent strategy may still fall for the off-centre effect because they would have to stick to the provisional decision made before or at the start of the run- up. In sum, in competitive situations a penalty taker’s liability for the off-centre effect is probably a function of both the goalkeeper’s and the penalty taker’s strategy.

The current study set out to test this hypothesis in a representative task design. To this end, penalty takers were required to position the goalkeeper in the middle of the goal before they took the penalty kick. No instructions or hints regarding the side to which to kick were given. This guaranteed that the penalty takers consciously perceived the goalkeeper standing in the middle of the goal, while in all likelihood the goalkeeper would be positioned marginally off-centre (people are generally poor in accurately indicating the middle of a line [16]). If the goalkeeper was indeed not positioned in the exact centre of the goal, the goalkeeper’s distance from the centre of the goal could be considered too small to be consciously perceivable. This is not to say, however, that it cannot affect kick decisions of the penalty takers implicitly (if the distance does not drop below the objective perceptual threshold of perception, which demarcates stimuli that are too weak to influence decision-making from stimuli that are strong enough to have an impact on decision-making, but without becoming consciously aware). Consequently, we hypothesised that if the off-centre effect actually translates to a more representative real-life situation, penalty takers should choose to kick the ball to the side with more space more often than to the side with less space, if they erroneously perceived the goalkeeper to stand in the exact middle of the goal. In addition, penalty takers were asked to either follow a keeper dependent or a keeper independent strategy, while goalkeepers were told to either dive before or after ball contact. We hypothesised that penalty takers would be liable to the off-centre effect if they adopted a keeper independent strategy. For the keeper dependent strategy, however, we hypothesised that penalty takers would only be liable to the effect in case a goalkeeper decided to dive late. When goalkeepers dive early, any implicit influence of information about keeper position that may affect decisions on kick directions were expected to be overruled by more salient information from diving direction. Kicks would then be directed to the opposite side of the goalkeeper’s dive, rather than the side with greater space (before the goalkeeper moved).

## Methods

### Participants

This study was conducted according to the principles expressed in the Declaration of Helsinki. With the written approval of the German Sport University’s ethics committee (statement that informed consent and local ethics committee approval has been provided for human studies) fifty intermediate skilled male right-footed soccer players with an average age of 18.7 years (SD = 2.1) were recruited to take part. All participants have been playing amateur soccer on a regular basis for a minimum of 8 years and reported that they routinely took penalty kicks during soccer training. In addition, two intermediate skilled male right-footed amateur goalkeepers (both 18 years) participated in the study.

### Materials and Procedure

The experiment took place on an adult-sized soccer field. The goal’s dimensions (7.32 m x 2.44 m), the distance between penalty mark and goal centre (11m) and the ball size (size 5) were in accordance with FIFA rules. The goal stood in front of a background with minimum visual texture.

First, participants provided written informed consent and completed a questionnaire asking for personal details. Subsequently, penalty takers read an instruction on how to use the two penalty kick strategies. Instructions for the keeper dependent strategy read as follows: “Direct the penalty kick to the empty side of the goal by anticipating the movement direction of the keeper. Wait as long as possible to choose a side in the case you are approaching the ball and the keeper does not move.” Instruction for the keeper independent strategy read: “Choose a side before starting the run-up and direct the penalty kick towards that side regardless of the keeper’s actions”. After reading the instructions they used a laser pointer from a position four steps behind the penalty mark (from where they were supposed to start the run-up) to indicate the centre of the goal. This position was marked on a measuring tape that was placed on the goal line and subsequently goalkeepers had to position themselves with their mid-sagittal plane directly above this mark. Penalty takers were explicitly informed that goalkeepers returned to this position for every of the following penalty kicks. One goalkeeper was trained and instructed to commit to one side of the goal before foot-ball contact (i.e., to prioritize timing), while the second goalkeeper was trained and advised to wait until foot-ball contact before diving (i.e., prioritize direction). We adopted this procedure because of the concern that goalkeepers would fall back to their habitual routines adjusting their actions to the penalty taker and/or mixing goalkeeping strategies if instructed to prioritize timing for some kicks and direction in others. (Admittedly, with this procedure some representativity was sacrificed to enhance experimental rigor). Goalkeepers were told to assume an arms-parallel posture before jumping to the ball in order to avoid any influence of posture on perception [17].

Half of the penalty takers took 20 penalty kicks against the goalkeeper who was instructed to dive early. The other half of participants also took 20 penalty kicks but faced the goalkeeper who dived late. Penalty takers, however, were not informed about the goalkeeper’s instructions. The penalty takers were advised what penalty kick strategy to use before each single kick. The penalty taker followed each of the two strategies 10 times in a randomized order. A research assistant who was naïve to the purpose of the experiment noted whether the penalty kicks were directed to the bigger side of the goal and whether the goalkeepers adhered to instructions regarding movement onset. (Only kicks in which goalkeepers acted according to the instruction regarding movement onset were included for analysis.)

### Analyses

Percentages of kicks to the side with greater space were calculated for each of four combinations of goalkeeper and penalty kick strategies and submitted to a 2 (goalkeeper strategy: early, late) x 2 (penalty take strategy: keeper dependent, keeper independent) ANOVA with repeated measures on the last factor. To verify that selection of the side with greater space was above chance, one-sample t-tests for each of four combinations of strategies were performed.

## Results

**Fig 1:**
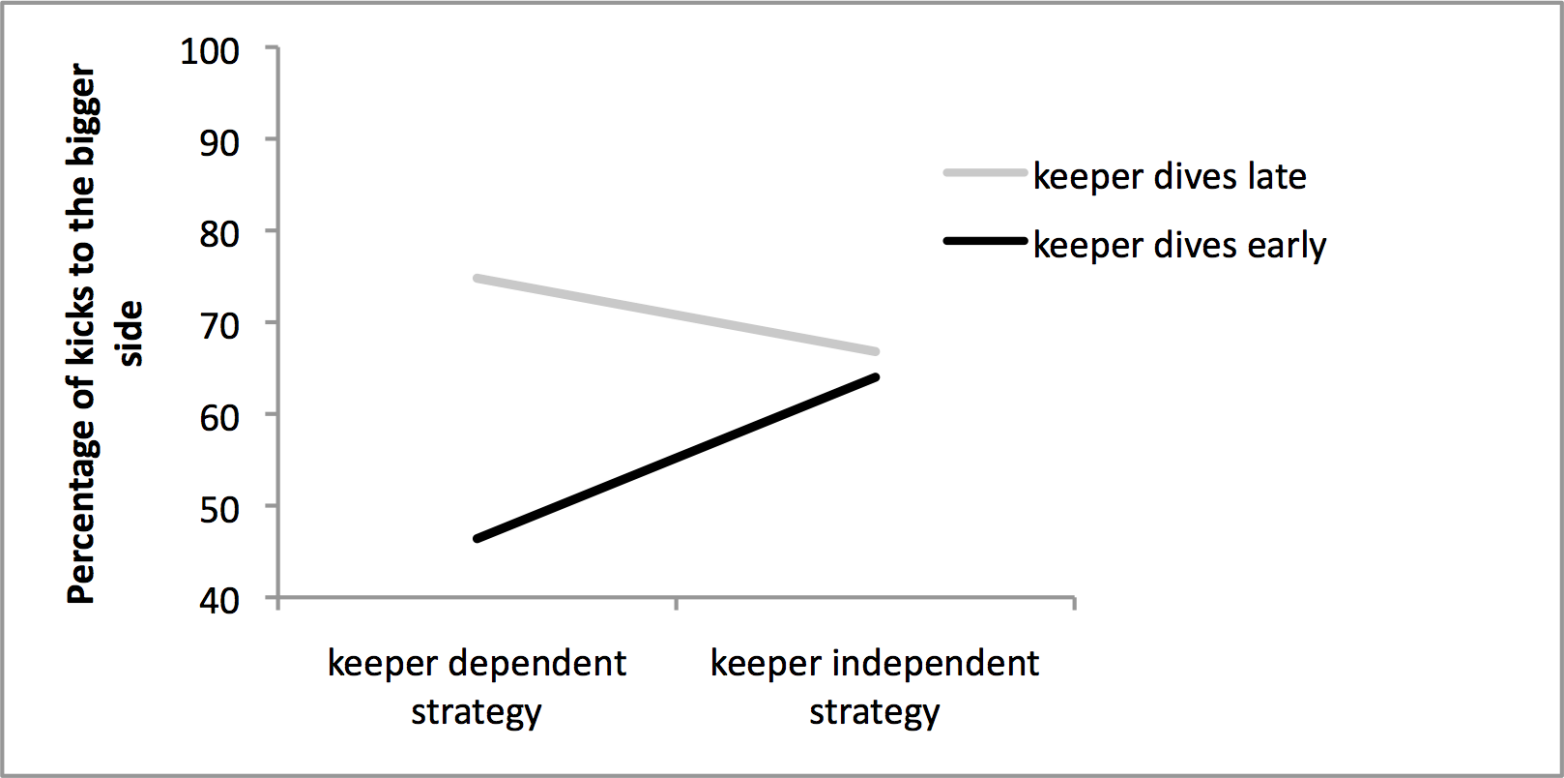
Kicks to the bigger side of the goal as a function of goalkeeper and penalty kick strategy.

No penalty takers placed the goalkeeper in the exact centre of the goal. On average the goalkeeper was positioned 3.5 cm (SD: 7.7) to the right. Fig 1 shows that the percentage of kicks to the side with greater space was influenced by a combination of the two strategies. This was confirmed by a significant main effect for goalkeeper strategy, *F* (1, 48) = 7.81, *p* < .01, *η* ^*2*^ = .14, and a significant interaction between penalty kick and goalkeeper strategy, *F* (1, 48) = 7.04, *p* < .02, *η* ^*2*^ = .13. Bonferroni corrected pairwise comparisons showed that the percentage of kicks to the side with greater space was higher when a goalkeeper acted late compared to early, but only when the penalty taker had adopted a keeper dependent strategy. When a keeper independent strategy was used no such difference was found (all *p*’s < .004). In other words, the percentage of kicks to the side with greater space was lower for the combination of a keeper dependent strategy and early diving goalkeeper strategy than for any other combination of strategies. Subsequently, Bonferroni corrected one-sample t-tests showed that the percentage of kicks to the side with greater space did not exceed 50% for the combination of keeper dependent strategy and early diving goalkeeper strategy, *t*(24) = .834, *p* > .4, one-tailed. For the other combinations, the percentage of kicks to the side with greater space did significantly exceed 50%, *t*’s(24) > 2.41, *p*’s < .025 (one-tailed), which indicates that the off-centre effect did occur.

## Discussion

The current study scrutinized whether implicitly perceived displacements of a goalkeeper relative to the goal centre also influence a penalty taker’s decisions for kicking direction in real-life environments that are more representative for competitive penalty kicking than the picture-based environments in previous work. In particularly, we asked whether the occurrence of the off-centre effect depends on the strategies that goalkeepers and penalty takers employ. Results showed that the goalkeeper’s position also affects a kicker’s choice for kick direction implicitly in situations that are more representative for competitive penalty kicks. This was generally true when the penalty kicker adopted a keeper independent strategy, but also when they used a keeper dependent strategy and the goalkeeper waited long. However, the off-centre effect dissolved when the penalty taker took the actions of the goalkeeper into account *and* the goalkeeper dived early. We can be sure that the current penalty takers were truly unaware because they had indicated the position where the goalkeeper stood as being exactly in the middle. Therefore, perception of the marginal displacement from the centre must have been below the subjective and above the objective threshold. Hence, even though there were several other factors that may have influenced decisions of kick directions deliberately (e.g., prior success kicking to a certain side, natural kicking side, goal keeper preference [18]) implicit perceptions of the goalkeeper’s position still affected the kick direction. However, it cannot be ruled out that the off-centre effect can be reduced or cancelled out by factors that did not play a role in our study. Considering that anxiousness affects penalty takers’ attentional control [19] (i.e., penalty takers tend to attend to other more conspicuous stimuli when anxious) the effects of implicit perception on decision-making may diminish with higher levels of anxiety (as present in real competitions). Likewise with more skilled players the effect may or may not be less pervasive. This remains to be addressed for future research. This is important because to the best of our knowledge there is no systematic analysis of concurrent implicit and explicit perceptions on decision-making. For instance, subliminal priming and attentional blink paradigms typically present stimuli either above or below subjective threshold, but not at the same time. The current results tentatively suggest that implicit perception can preserve its potential for influencing decision-making even when other stronger, more enduring and consciously accessible competing information sources are available [20, 21].

Our study underscores that also in experimental designs that involve a real goalkeeper who tries to save the ball, a goalkeeper can influence the penalty taker’s goal side selection by simply placing himself marginally off-centre. Doing so significantly enhanced the chance of the ball being directed to the side of the goal with greater space. The off-centre effect was quite pervasive, but did not last when penalty takers used a keeper dependent strategy and goalkeepers committed to one side sufficiently early to allow penalty takers to kick the ball to the opposite side. Most likely, any effect of goalkeeper position on the goal line before the run-up was overridden by the more conspicuous and explicit perception that the goalkeeper jumps to one particular side with sufficient time available to actually adjust kick direction accordingly. This is probably not surprising with an early diving goalkeeper. However, it is important to realize that in competition this combination of penalty taker and goalkeeper strategies does not occur very often: only an estimated 10–15% of penalty kicks are taken with a keeper-dependent strategy [14] and better goalkeepers tend to wait relatively long before diving [6, 10, 11]. It is remarkable, however, that the off-centre effect is preserved for all other combinations of strategies, the more so because it is known that even with a keeper independent strategy the kicking accuracy is affected by the presence of a goalkeeper [22]. This suggests that the effect is produced prior to the run-up. Consequently, for penalty takers to make sure that they choose kick direction fully independent of a goalkeeper’s actions, they should select side (or corner) before they approach the penalty box, or even before the game. Deciding before the walk to the penalty box has also been associated with more effective problem-focussed coping strategies to manage the stressful situation [23]. In fact, also penalty takers who adopt a keeper dependent strategy should be advised to make the provisional decision on kick direction before walking to the penalty box. Conversely, goalkeepers increase the chance that their position relative to the middle of the goal line impacts the penalty takers’ kick direction by waiting long. In the current study we advised goalkeepers to move after foot-ball contact. However, penalty takers take at least 200–250 ms (and probably more) to alter kick direction. Hence, goalkeepers can probably start moving before penalty takers kick the ball without annihilating the off-centre effect, irrespective of the strategy that the penalty taker employs.

To sum up, we showed that a goalkeeper who stands marginally off-centre can also influence kick direction unbeknownst to penalty takers in more representative task designs in which both the goalkeeper and the penalty taker act strategically. That is, unless the penalty taker uses a keeper dependent strategy and sees the goalkeeper commit early, the goalkeeper position can implicitly affect kick direction (if the penalty taker hasn’t chosen to which side to kick before walking to the penalty box).

## Perspectives

Two main goals remain for sport related research. First, it needs to be addressed in future research if goalkeepers can really increase the number of penalty kick saves if they record a penalty taker’s preferred penalty kick strategy and prioritize time or direction of dive accordingly, in particular goalkeepers who experience difficulties anticipating based on the kicker’s action. It further seems worthwhile to identify other situations in sports in which an opposing player’s decision-making can be influenced by marginally changing someone’s position. In this regard, positions of receiving players in volleyball or tennis may affect the direction of an upcoming serve. The argumentation in the current and previous studies [1] is that the off-centre effect is a case of implicit perception. If the effect is indeed triggered by implicit perception of a goalkeeper’s displacement a comparable effect of any displacement that is relevant for decision-making should be provable (e.g. more services to the right side if a returning player is marginally displaced to the left).

## Supporting Information

S1 Table Data set consisting of individual means for all variables. (XLSX)
